# Trends and Innovations in Autologous Breast Reconstruction

**DOI:** 10.1055/s-0043-1767788

**Published:** 2023-05-29

**Authors:** Nicole E. Speck, Vendela Grufman, Jian Farhadi

**Affiliations:** 1 Plastic Surgery Group, Zurich, Switzerland; 2 Department of Plastic, Reconstructive, Aesthetic and Hand Surgery, University Hospital Basel, Basel, Switzerland; 3 University of Basel, Basel, Switzerland

**Keywords:** microsurgery, mammaplasty, perforator flap, innovation, technology

## Abstract

More than 40 years have passed since the description of the first “free abdominoplasty flap” for breast reconstruction by Holmström. In the meantime, surgical advances and technological innovations have resulted in the widespread adoption of autologous breast reconstruction to recreate the female breast after mastectomy. While concepts and techniques are continuing to evolve, maintaining an overview is challenging. This article provides a review of current trends and recent innovations in autologous breast reconstruction.

## Introduction

Fifty years after the description of the first clinical free flap transfer by McLean and Buncke and more than 40 years after the first “free abdominoplasty flap” for breast reconstruction by Holmström, autologous breast reconstruction has become the gold standard for recreating the female breast after mastectomy. 1 2

In many centers, the goal of autologous breast reconstruction has transitioned past flap success to maximizing the aesthetic result and patient satisfaction while minimizing complications. 3 This shift has become possible thanks to new concepts, innovations in technique, and technological advances. 4

While concepts and techniques are continuing to evolve, maintaining an overview is challenging. The aim of this article is to provide a concise overview of current trends and recent innovations in autologous breast reconstruction.

## Methods

**Fig. 1:**
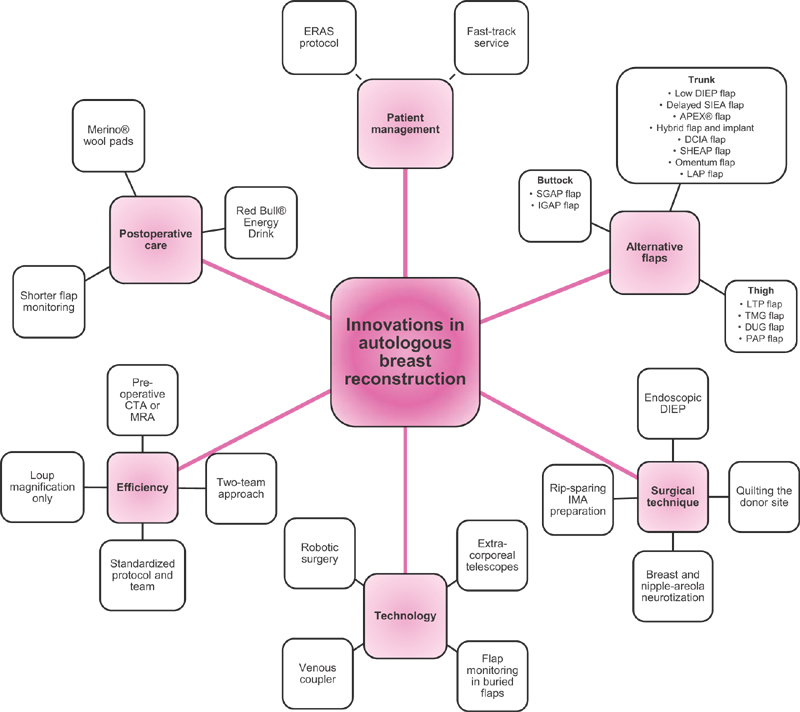
Mind map providing an overview of innovations in autologous breast reconstruction. APEX, abdominal perforator exchange; CTA, computed tomographic angiography; DCIA, deep circumflex iliac artery; DIEP, deep inferior epigastric perforator; DUG, diagonal upper gracilis; ERAS, enhanced recovery after surgery; IGAP, inferior gluteal artery perforator; LAP, lumbar artery perforator; LTP, lateral thigh perforator; MRA, magnetic resonance imaging angiography; PAP, profunda artery perforator; SGAP, superior gluteal artery perforator; SHAEP, stacked hemiabdominal extended perforator; TFL, tensor fasciae latae; TMG, transverse myocutaneous gracilis.

As many new concepts and innovations are presented at conferences before appearing as written publications, we screened conference abstracts from previous London Breast Meetings to achieve an overview of the most recent trends. Abstracts from 2015 to 2022 were screened for content related to trends or innovations in autologous breast reconstruction. Forty eligible contributions were identified in the conference programs over the course of the study period. All eligible contributions were then searched on the electronic database “Aesthetic and Reconstructive Breast Surgery Network” (ARBS Network, Copyright 2022 Mark Allen Group, United Kingdom). For 25 contributions, an on-demand video was available on ARBS Network. After viewing, the contributions were grouped into key areas in the preoperative, intraoperative, and postoperative setting. An ordered list of all contributions is provided in Table 1 . For all contributions with a hyperlink provided, the video is available on demand for the readers. More papers related to the content viewed were then searched on the electronic database MEDLINE (Bethesda, MD: U.S. National Library of Medicine). Fig. 1 provides a concise overview of various innovations.

**Table 1:** Ordered list of contributions regarding innovations in autologous breast reconstruction presented at London Breast Meetings 2015–2022

Topic	Title	Presenter	Year	Web link	Reference
Alternative flaps	Latest advances in autologous flaps for thin patients: LTP flap	Stefania Tuinder	2015	https://arbsnetwork.com/videos/latest-advances-in-autologous-flaps-for-thin-patients-pap-lap-ltp-dcia-stefania-tuinder-koenraad-van-landuyt-ed-buchel-c5fa334e-6590-49ff-b9eb-31c74d3b920b	28
Alternative flaps	Latest advances in autologous flaps for thin patients: TFL/LAP flap	Koenraad van Landuyt	2015		30
Alternative flaps	Latest advances in autologous flaps for thin patients: DCIA flap	Ed Buchel	2015		
Alternative flaps	The low DIEP flap	Jinsup Eom	2017	https://arbsnetwork.com/videos/innovation-in-autologous-flaps-the-low-diep-flap-jinsup-eom	19
Alternative flaps	Stacked LTP flap	Robert Allen	2017	https://arbsnetwork.com/videos/innovation-in-autologous-flaps-lf-flap-bob-allen	
Alternative flaps	APEX flap	Frank DellaCroce	2017	https://arbsnetwork.com/videos/innovation-in-autologous-flaps-apex-flap-frank-della-croce	18
Alternative flaps	Omentum flap	Peter Sandbichler	2017	https://arbsnetwork.com/videos/innovation-in-autologous-flaps-omentum-flap-peter-sandbichler	32
Alternative flaps	DUG flap	Joseph Dayan	2017	https://arbsnetwork.com/videos/innovation-in-autologous-flaps-dug-flap-joseph-dayan	57
Alternative flaps	Boom flaps: hybrid autologous reconstruction	Suhail Kanchwala	2017	https://arbsnetwork.com/videos/boom-flaps-hybrid-autologous-reconstruction-suhail-kanchwala	20
Alternative flaps	SHAEP flap	Joshua Levine	2021		17
Alternative flaps	Surgical delay of the DIEP flap	Robert Allen Sr.	2021		21
Surgical technique	Endoscopic DIEP flap	Michael Atlan	2017	https://arbsnetwork.com/videos/innovation-in-autologous-flaps-endoscopic-diep-flap-michael-atlan	35
Surgical technique	Time to stop rib resection?	Mihye Choi	2019	https://arbsnetwork.com/videos/65059-time-to-stop-rib-resection-in-autologous-reconstruction-mihye-choi	37
Surgical technique	To quilt or not to quilt the donor site	Susana Correa	2019	https://arbsnetwork.com/videos/65060-to-quilt-or-not-to-quilt-the-donor-site-in-autologous-reconstruction-susana-correa	
Surgical technique	Avoiding donor site morbidity after DIEP flap: the abdominoplasty free flap	Moustapha Hamdi	2021		25
Surgical technique	Breast and nipple-areolar neurotization	Koenraad van Landuyt	2021		
Surgical technique	The sense and nonsense of flap neurotization	Koenraad van Landuyt	2022		
Technology	Robotic surgery in breast and microsurgery	Benjamin Sarfati	2017	https://arbsnetwork.com/videos/robotic-surgery-in-breast-and-microsurgery-benjamin-sarfati-marco-innocenti-jesse-selber	58
Technology	Robotic surgery in breast and microsurgery	Marco Innocenti	2017		45
Technology	Robotic surgery in breast and microsurgery	Jesse Selber	2017		44 59 60
Technology	Extracorporeal telescopes in microsurgery	Edward Chang	2021		47
Technology	Flap monitoring in buried flaps	Mark Ho Asjoe	2022		
Technology	Advances in microscopes and robotics	Edward Chang	2022		
Efficiency	Tips and tricks in the efficiency process	Liza Wu	2017	https://arbsnetwork.com/videos/tips-and-tricks-in-the-efficiency-process-for-autologous-breast-reconstruction-liza-wu	
Efficiency	Efficient raising of the DIEP flap	Frank DellaCroce	2017	https://arbsnetwork.com/videos/efficient-raising-of-the-diep-flap-frank-dellacroce-venkat-ramakrishnan	
Efficiency	Efficient raising of the DIEP flap	Venkat Ramakrishnan	2017		61
Efficiency	Training in microsurgery: breeding the efficient microsurgeon	Takumi Yamamoto	2022		
Postoperative care	Postoperative wool pads instead of forced-air warming blankets post microsurgical procedures	Welmoed Keijzer	2018	https://arbsnetwork.com/documents/wool-pads-instead-of-forced-air-warming-blankets-post-microsurgical-procedures	52
Postoperative care	Flap monitoring: short and sweet	Sinikka Suominen	2019	https://arbsnetwork.com/videos/65061-flap-monitoring-in-autologous-reconstruction-short-and-sweet-sinikka-suominen	
Postoperative care	Updates on flap monitoring	Chris Andree	2021		
Postoperative care	Flying high – effect of Red Bull Energy drink	Nicole Speck	2021		
Patient management	Fast track autologous reconstruction service	Mark Smith	2018	https://arbsnetwork.com/videos/66766-fast-track-autologous-reconstruction-service-mark-smith-christian-bonde	
Patient management	Fast track autologous reconstruction service	Christian Bonde	2018		5 56
Patient management	Enhanced recovery: is it worthwhile?	Joan Lipa	2019	https://arbsnetwork.com/videos/65066-enhanced-recovery-in-autologous-reconstruction-is-it-really-worthwhile-joan-lipa	
Patient management	Day case free flaps	Adam Blackburn	2022		

Abbreviations: APEX, abdominal perforator exchange; DCIA, deep circumflex iliac artery; DIEP, deep inferior epigastric perforator; DUG, diagonal upper gracilis; LAP, lumbar artery perforator; LTP, lateral thigh perforator; SHAEP, stacked hemiabdominal extended perforator; TFL, tensor fasciae latae.

## Results

### Preoperative Setting

#### Patient Management

Enhanced recovery after surgery (ERAS) protocols have been successfully implemented in autologous breast reconstruction. 5 6 In the preoperative setting, these protocols include detailed patient education and expectation setting by the surgeon and a certified breast reconstruction nurse. For this purpose, standardized information sheets or audio-recordings have proven helpful. 7 As to nutrition, preoperative carbohydrate loading with maltodextrin-based drinks has been shown to slightly reduce length-of-stay (LOS) without increased adverse events when compared with fasting or placebo. 8

### Intraoperative Setting

#### Efficiency

Several strategies have been developed to optimize efficiency in autologous breast reconstruction. In a prospective study, the use of preoperative computed tomographic angiography was associated with decreased operative times in deep inferior epigastric perforator (DIEP) flap reconstruction, specifically concerning perforator identification and perforator selection. 9 A cosurgeon approach has been shown to reduce operative time, average LOS, and postoperative complications in a retrospective study. 10 In another retrospective review of 104 DIEP flaps where standardized preoperative planning, operating room (OR) setup, and operative technique were applied, the average operative times were as short as 3 hours and 21 minutes for a unilateral DIEP and 5 hours and 46 minutes for a bilateral DIEP. 11 The authors' standardized protocol also included a dedicated OR team with staff members remaining in the room during the length of the procedure to minimize transitions of care. Using process mapping and analysis, Haddock and Teotia furthermore identified eight critical maneuvers which could maximize efficiency and safety for DIEP flap reconstruction. 12

On a technical note, performing flap dissection and the anastomosis under loupe magnification without the use of a microscope may speed up the operative process by providing more space for simultaneous mastectomy on the contralateral side while performing an anastomosis. 13 Moreover, the venous coupler has been shown to significantly reduce operation time compared with a hand-sewn anastomosis. 14

#### Alternative Flaps

The trend for perforator flaps has been continuing ever since the landmark publication about the first perforator flap by Koshima and Soeda in 1989. 15 In 2014, Healy and Allen evaluated 20 years of performing perforator flaps in breast reconstruction, concluding that the DIEP flap has remained the first choice. 16 Over time, multiple variations of the abdominally based flap have been developed. For patients with insufficient abdominal tissue requiring bilateral autologous breast reconstruction, the stacked hemiabdominal extended perforator is an excellent choice. 17 This bipedicled flap is designed as a combination of the DIEP and a second, more lateral pedicle: the deep or superficial circumflex iliac perforator vessels, the superficial inferior epigastric artery (SIEA), or a lumbar artery or intercostal perforator. In cases where anatomical variations in perforator arrangement might impair the surgeon's ability to effectively avoid transection of the rectus muscle or nerve structure, the abdominal perforator exchange (APEX) flap has been shown to be a safe choice. 18 The low DIEP can be used to reconstruct moderately sized breasts if reliable perforators exist below the umbilicus, offering the advantage of a low scar close to the pubic rim and obviating the need for umbilical detachment. 19 In case of insufficient abdominal tissue, a hybrid approach may be used, combining a pre-pectoral silicone gel implant with a DIEP flap. 20 The SIEA flap allows autologous breast reconstruction without violating the rectus fascia. While 6 to 70% of SIEAs are less than 1.5 mm in diameter and therefore considered unreliable, surgical delay of the SIEA flap has been shown to increase SIEA diameter, thus increasing the reliability of this flap for breast reconstruction while reducing abdominal morbidity. 21

However, some patients might not be amenable to an abdominally based flap due to lack of volume or previous surgery. 22 For this subset of patients, several alternative donor sites can be offered. 23 On the thigh, these include the transverse myocutaneous gracilis (TMG), the diagonal upper gracilis (DUG), the profunda artery perforator (PAP), and the lateral thigh perforator (LTP) flap. 23 The TMG flap is the most used alternative flap for breast reconstruction. 24 Disadvantages include the limited amount of skin and soft tissue available, relatively short pedicle, and risk of wound dehiscence and scar migration. 25 The DUG flap offers a safe alternative to the TMG flap by increasing the amount of skin and fat available and allowing optimal wound healing due to its flap design along Langer's lines. 26 The PAP flap offers several advantages including large vessels with consistent anatomy, a long pedicle, and a muscle-sparing alternative to the gracilis-based flaps. 27 Alternatively, the LTP flap is a good option to reconstruct small to medium sized breasts in patients with a “saddlebag” deformity. 28 On the buttock, the superior gluteal artery perforator and the inferior gluteal artery perforator flap can be harvested. 29 The lumbar artery perforator flap is another valuable alternative flap. 30 It is considered superior to the DIEP flap in mimicking the shape and feel of native breast due to the quality of the lumbar fat and the gluteal extension. 31

Furthermore, laparoscopically harvested omental flaps have been proposed to reduce donor site morbidity. 32 Most recently, flap harvest has been achieved through a single port. 33 Lastly, partial or total breast reconstruction can be achieved with pedicled perforator flaps from the lateral thoracic area. 34 Flap types include the thoracodorsal artery perforator and the lateral intercostal artery perforator flap.

#### Surgical Technique

To minimize donor site morbidity, Stroumza et al have proposed dissecting perforators endoscopically using pediatric instruments. 35 A laparoscopic approach to flap harvest has been associated with an even shorter fascial incision length compared with the endoscopic approach in another center. 36

To reduce intra- and postoperative pain and to prevent thoracic contour deformities, some authors routinely dissect the internal mammary vessels without rib resection. 37 38 This technique seems to be feasible in most cases, except for situations where greater vessel exposure is needed. 38

As to donor site closure, several authors have advocated the use of barbed progressive tension sutures either on their own or in combination with suction drains. 39 40 The use of barbed progressive tension sutures on their own has not been associated with higher seroma rates or wound dehiscence and may promote patient mobility and increase satisfaction. 40 Visconti et al have furthermore combined progressive high-tension sutures with cannula-assisted lipectomy and limited flap undermining (“CALP” technique) to achieve aesthetic closure of the DIEP flap donor site. 41 This technique was associated with significantly lower daily drainage output, fewer donor site complications, and better skin sensibility compared with the control group who received traditional abdominoplasty closure.

Lately, neurotization has gained increased attention in autologous breast reconstruction. 42 While existing data is heterogeneous, neurotization may restore sensation earlier and at lower stimulation thresholds. 43

#### Technology

To reduce donor site morbidity, the robotic DIEP flap has been developed. It allows maximum pedicle length while limiting fascial incision to 1.5 to 3 cm. 44 Robotic technology has also been implemented to perform anastomoses. Two robots for microsurgery exist: MUSA by Microsure (Microsure B.V., Eindhoven, Netherlands) and Symani by MMI (Medical Microinstruments, Inc., Wilmington, DE). 45 This technology aims at increasing surgical precision by eliminating tremor and allowing access from various angles. 46

Recently, exoscopes have emerged as alternatives to surgical loupes and traditional operating microscopes for surgical magnification. Theoretical advantages of the exoscope over conventional devices include improved surgeon ergonomics, superior three-dimensional, high-definition optics, and greater ease-of-use. 47

Furthermore, indocyanine green fluorescence angiography is useful to evaluate flap perfusion before selecting a perforator and to prevent eventual fat necrosis by visualizing relatively underperfused flap tissue. 48 When assessing mastectomy skin flaps it may be a helpful tool to decide if mastectomy skin should be excised and replaced with donor site skin to prevent mastectomy skin flap necrosis. 49

### Postoperative Setting

#### Postoperative Care

Regional blocks have received increasing popularity to reduce postoperative pain and analgesic load at the donor site and recipient site. 50 This has been shown to decrease postoperative opioid consumption and decrease LOS. 51

Warming of the recreated breast with preshaped Merino wool pads has been shown to be a safe alternative to traditional heating blankets. 52 The wool pads provide the advantage of selective warming of the breast without overheating of the body, avoid a bulky machine and allow continued warming after hospital discharge.

To reduce the postoperative need for vasopressors and intravenous volume administration, the effect of Red Bull Energy drink has been investigated. It has been associated with an increase in systolic blood pressure while having a diuretic effect when administered on the day of surgery and postoperative day (POD) 1. 53

#### Patient Management

ERAS protocols have allowed for “fast track” autologous reconstruction. Considering that very few flaps are salvaged after POD 2, a trend has emerged to discharge patients earlier. 54 Some authors have performed breast reconstruction as an outpatient procedure with discharge as early as 23 hours postoperatively. 55 This has not been associated with an increased flap loss rate. 56 Of note, the whole team including nursing staff needs to emphasize these goals. The “fast track” service is further facilitated by standardized postdischarge planning.

## Discussion and Conclusion

This article provides a concise overview of current trends and recent innovations in autologous breast reconstruction. This review has some strengths. By sourcing data from previous London Breast Meetings, the authors could identify hitherto unpublished results. Also, the videos available for many contributions might provide valuable information for the interested reader. However, while many state-of-the-art trends could be identified by screening recent conference abstracts, this review is not complete. Identifying all possible innovations as part of a systematic review was beyond the scope of this article but could be part of a future research project. Furthermore, we did not aim at providing detailed descriptions of the different innovations. More information can be found in the referenced literature or web links provided.

Thanks to numerous innovations, autologous breast reconstruction has become the gold standard to recreate the female breast after mastectomy. As new concepts and techniques continue to evolve, the focus of autologous breast reconstruction is transitioning past flap success to increasing patient satisfaction.
